# High-throughput sequencing identified circular RNA circUBE2K mediating RhoA associated bladder cancer phenotype via regulation of miR-516b-5p/ARHGAP5 axis

**DOI:** 10.1038/s41419-021-03977-1

**Published:** 2021-07-20

**Authors:** Chen Yang, Zezhong Mou, Siqi Wu, Yuxi Ou, Zheyu Zhang, Xinan Chen, Xiyu Dai, Chenyang Xu, Shanhua Mao, Haowen Jiang

**Affiliations:** 1 Department of Urology, Huashan Hospital, Fudan University, Shanghai, China; 2 Fudan Institute of Urology, Huashan Hospital, Fudan University, Shanghai, China; 3 National Clinical Research Center for Aging and Medicine, Fudan University, Shanghai, China

**Keywords:** Non-coding RNAs, Bladder cancer

## Abstract

Bladder cancer (BC) is known as a common and lethal urinary malignancy worldwide. Circular RNAs (circRNAs), an emerging non-coding RNA, participate in carcinogenesis process of several cancers including BC. In this study, high-throughput sequencing and RT-qPCR were applied to discover and validate abnormal high expression of circUBE2K in BC tissues. Fluorescence in situ hybridization (FISH) was used to detect hsa_circ_0009154 (circUBE2K) expression and subcellular localization in BC tissues. High circUBE2K predicted unfavorable prognoses in BCs, as well as correlated with clinical features. CCK8, transwell, EdU and wound healing assays demonstrated down-regulating circUBE2K decreased BC cell phenotype as proliferation, invasion, and migration, respectively. Further studies showed that circUBE2K promoted BC progression via sponging miR-516b-5p and enhancing ARHGAP5 expression through regulating RhoA activity. Dual-luciferase reporter, FISH and RNA pulldown assays were employed to verify the relationships among circUBE2K/miR-516b-5p/ARHGAP5/RhoA axis. Down-regulating miR-516b-5p or overexpressing ARHGAP5 restored RhoA activity mediated BC cell properties after silencing circUBE2K. Subcutaneous xenograft and metastasis model identified circUBE2K significantly increased BC cell metastasis and proliferation in-vivo. Taken together, we found that circUBE2K is a tumor-promoting circRNA in BC that functions as a ceRNA to regulate ARHGAP5 expression via sponging miR-516b-5p.

## Introduction

Bladder cancer (BC), accounting for over 200,000 mortality in 2018, is the most prevalent urinary malignancy worldwide [1]. In China, its mortality and morbidity also ranked first among urinary system cancers [2]. Although targeted therapies have been wildly used, the prognosis of BC remains unfavorable due to its probability of relapse and metastasis [3]. Therefore, it is important to discover the molecular mechanism that drives BC progression and recurrence.

Whole genome sequencing has revealed protein-coding genes comprise only 2% of human genomes [4]. However, large quantities of sequences are transcribed into noncoding RNAs, which play a critical role in the regulatory of cancers [5]. circRNAs are covalently closed single structure and lack of 5′ caps and 3′ poly(A) tails, and are regarded as biomarkers due to its stability, abundance and conservation [6]. Increasing evidence suggests that most of circRNAs execute biological functions by working as miRNA sponges and regulate circRNA/miRNA/mRNA axis [7]. Additionally, it has also been found that circRNAs could function as RNA splicing regulators, be translated into peptides or proteins and sponge RNA-binding proteins to modulate the expression of proteins [8]. Recently, more and more studies demonstrated that circRNAs could modulate gene expression by interacting with miRNAs and by regulating competing endogenous RNAs (ceRNA) network in BC. For example, circITCH could inhibit BC progression by sponging miR-17/miR-227 and regulating p21 as well as PTEN expression [9]. On the contrary, circDOCK1 could promote tumorigenesis and work as a risk biomarker through circINTS4/miR-131-3p/SOX5 axis in BCs [10]. Therefore, it is of urgent need to discover novel bladder cancer-related circRNAs and clarify their molecular functions and mechanisms in the growth and metastasis of BC.

Herein, high through-put RNA-seq was determined to identify a previously undescribed circRNA termed circUBE2K. circUBE2K is located on chromosome 4, and is obviously overexpressed in BC tissues and cell lines. Kaplan-Meier survival analysis showed that high expression level of circUBE2K correlated with poor survival in BC patients. Knockdown of circUBE2K inhibited BC cells proliferation, invasion and epithelial-mesenchymal transition (EMT) by impeding TGF-β and PI3K-AKT-mTOR pathways. In addition, we identified circUBE2K could sponge miR-516b-5p and thus promote ARHGAP5 expression. Moreover, we verified that ARHGAP5 could suppress RhoA activity in BC to further promote EMT process. Therefore, circUBE2K may serve as a biomarker aiding prognosis prediction and potential therapeutic target for BC patients.

## Materials

### Patient samples and RNA-seq

The study protocol was approved by the Ethics Committee of Huashan Hospital (Shanghai, China; approval no. KY2011-009. A total of 3 μg of total RNA was extracted followed with RNase R treatment from each sample to the Illumina VAHTS Library Prep Kit (Vazyme Biotech., Nanjing, China) to detect differentially expressed circRNA [11].

### Total RNA isolation and quantitive reverse transcriptase PCR (qRT-PCR)

Total RNA was isolated from the tumor tissues or cells using TRIzol Reagent (Thermo Fisher Scientific, Invitrogen). RNA was reverse transcribed into cDNA using SuperScript II Reverse Transcriptase (Invitrogen). Then qRT-PCR was performed using an AB7300 thermo-recycler (Applied Biosystems), using relative expression of GAPDH as reference gene [12, 13].

### Cell culture and infection

Bladder transitional cell carcinoma cell lines (RT4, UM-UC-3, T24, 5637, J82) and immortalized uroepithelium cell line (SV-HUC-1) were maintained with DMEM (Gibco, USA) supplemented with 10% fetal bovine serum (FBS; Gibco, USA). To knockdown circUBE2K, lentiviral vector carrying shRNA was designed. Lentiviral packaging and infection were achieved as previous work. Small interfering RNA and miRNA mimics were transfected into cells using Lipofectamine 2000 (Invitrogen) [11, 14].

### Cell proliferation assay

Cellular proliferation was assessed with Cell Counting Kit-8 (CCK8; Sigma-Aldrich, USA) according to the manufacturer’s procedure. For colony formation assay, cells were seeded into six-well plates at a density of 600 cells per well. After 7~9 days, cells were fixed and stained with crystal violet.

### Flow cytometry

Cells were isolated and stained for cell cycle (Cell Cycle Assay kit, Abcam, China). All samples were run on a BD LSRFortessa unit and analyzed by FlowJoX software (Flowjo LLC, Ashland, OR).

### RNase R treatment and actinomycin D assays

In total 5 × 10^5^ cells, T24 and UMUC-3 cells were isolated for total RNA and incubated 30 min at 37 °C of 5 U/μg RNase R (Epicentre Technologies, USA) and analyzation with qRT-PCR.

5 × 10^5^ cells T24 and UMUC-3 cells were seeded in six-well plates. 2 μg/ml Actinomycin D (Sigma) was added and normalized to vehicle treatment group.

### Transwell assay

Cell migration was analyzed using Transwell chambers (Corning, USA). Cells were cultured in serum-free DMEM with 5 µg/ml Mitomycin C in upper chamber to inhibit cell proliferation.

### Wound‐healing assay

Cells were seeded at the density of 30,000 cells/well and scrapped with pipette. A total of 5 μg/ml mitomycin C was supplemented in DMEM to inhibit cell proliferation. All wounds were observed 24 h after scraping.

### In situ hybridization and Immunohistochemistry

Tissue sections were incubated with digoxigenin-labeled RNA probe or antibody for further analysis [11, 14].

### Xenograft mouse model and metastasis model

For subcutaneous tumor formation assay, 1 × 10^7^ transfected T24 cells were subcutaneously injected (*n* = 6 for each group) and sacrificed 21 days after injection.

For metastasis analyses, 1 × 10^5^ transfected T24-luc cells were intravenously injected (*n* = 6 mice per group) and bioluminescence imaging after 28 days [15].

### Luciferase reporter assay

Wild type (WT) cDNA fragments with predicted hsa-miR-516b-5p binding site of circUBE2K and ARHGAP5 3′-untranslated region (3′-UTR) were recombined into psiCHECK-2 (Promega, Madison, WI, USA) for luciferase reporter assay [14].

### Western blot analysis

Lysates were prepared and separated by 10% SDS-PAGE and incubated with primary antibodies: anti-ARHGAP5, anti-N-Cadherin, anti-Vimentin, anti-RhoA, anti-pan-AKT, anti-P-AKT (Phospho Ser473), and anti-GAPDH (Abcam, Shanghai, China). After washing with PBST, the blots were incubated with HRP-conjugated IgG for 2 h. ECL substrate (CLiNX, Shanghai, China) were used for detecting HRP-conjugated antibody.

### RNA pulldown

Biotin-coupled circUBE2K probes and oligo probes were incubated with Streptavidin-coupled magnetic beads (Life Technologies, USA) for 3 h at room temperature to generate probe coated beads for analysis [16].

### RIP assay

RIP assay was performed using the Magnetic RIP RNA-binding protein immunoprecipitation kit (Millipore) [11].

### G-LISA assay

RhoA activity was measured using G-LISA Activation Assay Kits (Cytoskeleton). Briefly, cells were lysed for 15 min on ice after stimulation to centrifuge at 10,000×g for 1 min at 4 °C and assessed according to the manufacturer’s instructions.

### Bioinformatics analysis

miRanda [17], Circular RNA Interactome [18], and CircBank Database [19] were enrolled. For miRNA-gene interactions, four tools including miRDB [20], Encori [21], TargetScan [22], and miRWalk were used.

Time-dependent receiver operating characteristic (ROC) analysis was performed using R software (Version 3.6.2). Survival analysis was carried out by Kaplan-Meier Plotter [23], Gene expression profiling interactive analysis (GEPIA) [24]. Samples were divided by expression level of ARHGAP5 (based on median FPKM value) then proceeded to Gene Set Enrichment Analysis (GSEA) software [25].

### Statistical analyses

Statistical analyses were performed with GraphPad Prism 6.0 software (GraphPad) as indicated. Results were considered statistically significant when ^*^P < 0.05; ^**^P < 0.01; ^***^P < 0.001.

## Results

### Expression of circUBE2K was upregulated in BC and was related to prognosis

**Fig. 1:**
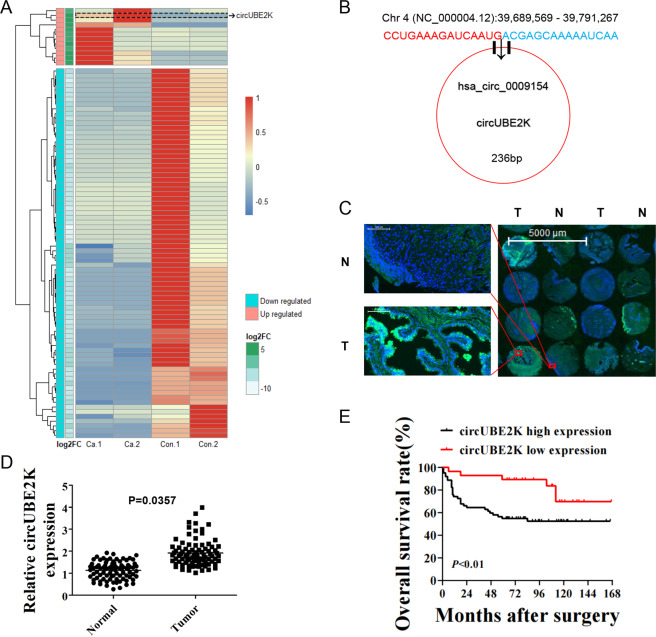
Expression of circUBE2K was upregulated in BC and was related to bladder cancer prognosis. **A** Heatmap illustrated normalized expression patterns of 90 differentially expressed circRNAs between two BC tissues and their paired adjacent normal tissues (*P*-value < 0.05 and |log2FC > 2). Each column represents one sample. Expression of circUBE2K was highlighted with a dashed frame. **B** The genomic location and illustration of has-circ-0009154. **C** Fluorescent in situ hybridization (FISH) for circUBE2K (Green) and DAPI (Blue) in 90 pairs of bladder cancer tissue microarray. circUBE2K is up regulated in BC tissue (Right panel) comparing to paired normal tissues (Left panel). Also, circUBE2K locates in BC cell cytoplasm. Representative area was selected. **D** Relative expression of circUBE2K in BC was higher than normal tissues (*P* < 0.05 versus normal tissue). **E** Kaplan-Meier plot for BC patients’ overall survival (OS), based on FISH circUBE2K expression level (*n* = 90; *P* < 0.01, Log rank test).

In order to identify biologically significant circRNA in BC, total RNA was extracted from two BC tissues and paired adjacent normal tissue (Fig. S1A). Differential gene expression analysis identified 12 upregulated and 78 downregulated circRNAs between normal and tumor tissues (Fig. S1B) (*P*-value < 0.05 and |log2FC | > 2) among which we identified circUBE2K as one of the most abnormal expressed circRNAs in 90 differential expressed circular RNA (Fig. 1A). Furthermore, we plotted genomic location and differential expression pattern of RNA-seq discovered bladder cancer-related circRNAs (Fig. S1C). The genomic location of circUBE2Kwas located in Chr 4(NC_000004.12):39689569–39791267, derived from gene UBE2K consist of 18320 bp while mature form of spliced circRNA is 236 bp (Fig. 1B).

Next, we carried out qRT-PCR across cell lines and observed higher relative circUBE2K expression in five bladder transitional cell carcinoma cell lines (RT4, UM-UC-3, T24, 5637, J82) compared to immortalized uroepithelium cell line (SV-HUC-1) (Fig. S2A). Although RNase R decreased linear UBE2K mRNA levels in UM-UC-3 and T24, it did not affect circUBE2K levels (Fig. S2B). Treatment by Actinomycin D in T24 and UM-UC-3 showed circUBE2K was stable in circular form in comparison to its lineal form, indicating circulation of the circUBE2K transcript (Fig. S2C). We further confirmed that circUBE2K was mainly located in cytoplasm and was upregulated in BC tissue by FISH and qRT-PCR (Fig. 1C, D). Kaplan–Meier plot showed that higher circUBE2K expression level correlated with lower overall survival (OS) rates (Fig. 1E) as well as tumor size, pathology stage, lymphatic metastasis, and tumor grade (Supplementary Table 1). In general, we suggested that circUBE2K played a vital role in BC oncogenesis and progression.

### Knockdown of circUBE2K suppressed BC cell proliferation and migration in vitro

**Fig. 2:**
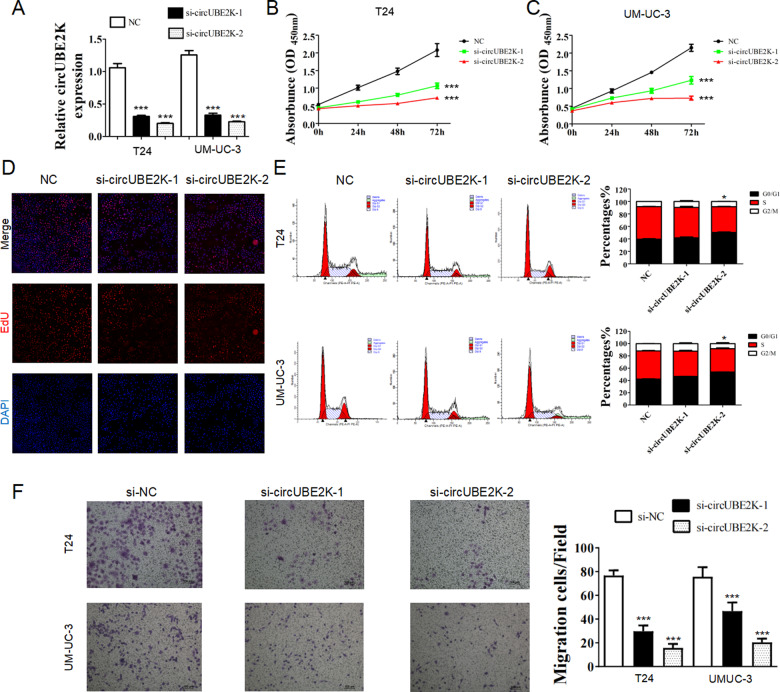
Knockdown of circUBE2K suppressed BC cell proliferation and migration in vitro. **A** Transfection of si-circUBE2K significantly reduced circUBE2K expression in T24 and UM-UC-3 (^***^P < 0.001 vs. NC). Error bars indicate SD. **B**, **C** CCK-8 assays showed that transfection of si-circUBE2K significantly inhibited proliferation of T24 (**B**) and UM-UC-3 (**C**). Error bars indicate SD (^***^P < 0.001 vs. NC). **D** EdU assay of T24 and si-circUBE2k transfected T24. **E** Cell cycle distribution of T24 and UM-UC-3 were analyzed by flow cytometry. **F** Transwell assay showed that circUBE2K knockdown hindered migration of T24 and UM-UC-3.

To explore the function of circUBE2K in BC cells, we designed three siRNAs targeting the back-splicing region of circUBE2K. Transfection of two si-circUBE2K significantly reduced circUBE2K expression in T24 and UM-UC-3 (Fig. 2A). CCK-8 assays showed that knockdown of circUBE2K significantly inhibited proliferation of T24 and UM-UC-3 (Fig. 2B, C), and the result was verified via EdU assay (Fig. 2D). Cell cycle distribution of T24 and UM-UC-3 were analyzed by flow cytometry. We observed elevated percentage of G0/G1 phase in si-circUBE2K-2 transfected T24 and UM-UC-3 cells (Fig. 2E). Transwell assay suggested that circUBE2K knockdown hindered migration of T24 and UM-UC-3 (Fig. 2F). Also, we identified such inhibitory phenotype (Fig. 2C–F) was associated with the decreased circUBE2K expression level (Fig. 2A) through different si-circUBE2K transfection, which further supported that knockdown of circUBE2K suppressed BC cell proliferation and migration in vitro. We selected si-circUBE2K-2 for further study.

### circUBE2K acted as a sponge for miR-516b-5p in BC cells

**Fig. 3:**
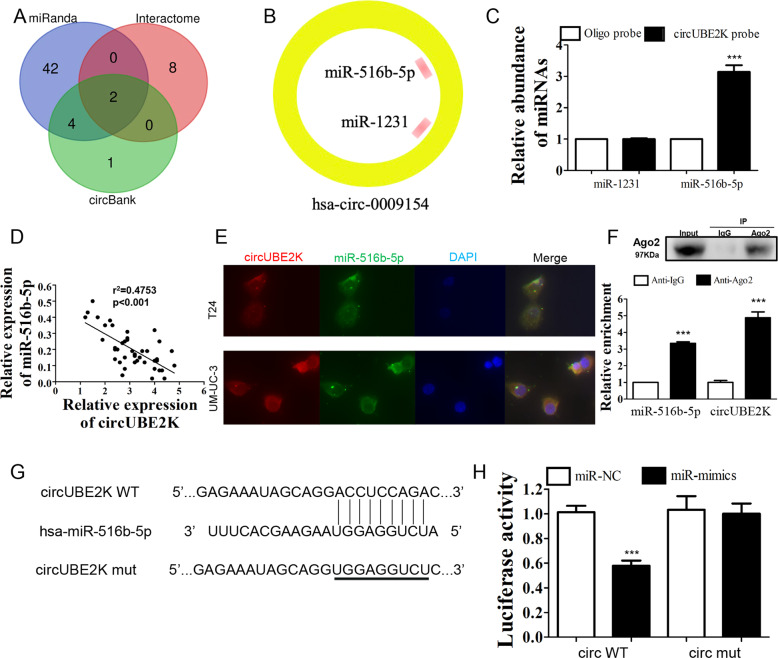
circUBE2K acted as a sponge for miR-516b-5p in BC cells. **A** Venn diagram showing overlapping of potential targets of circUBE2K predicted by miRanda, Circular RNA Interactome and CircBank Database. **B** Two miRNAs (miR-516b-5p and miR-1231) were predicted by three different prediction software programs. **C** RNA-pulldown assay followed by qRT-PCR suggested interaction between circUBE2K and miR-516b-5p. Error bars indicate SD (^***^P < 0.001 vs. other groups). **D** Scatter plot presented negative correlation between expression level of circUBE2K and miR-516b-5p in BC tissues (*n* = 42, *r*^2^ = 0.4753, *P* < 0.001). **E** Co-localization of circUBE2K (Red) and miR-516b-5p (Green). Nuclear staining was performed using DAPI (Blue). **F** RIP experiments were performed in T24 cells against IgG or Ago2, and the precipitated circUBE2K and miR-516b-5p were detected by qRT-PCR. ^***^P < 0.001 vs IgG. **G** Predicted binding site of circUBE2K and miR-516b-5p was demonstrated. The third row showed mutated circUBE2K sequence for further dual-luciferase reporter assay. **H** Luciferase activity were measured after co-transfection with circUBE2K (circWT)/mutated circUBE2K (circMUT) and miRNA mimics negative control (miR-NC)/miR-516b-5p mimics (miR-mimics) in HEK-293T cells. Error bars indicate SD (^***^P < 0.001 vs. vehicle).

Since circUBE2K was found to be predominately located in cytoplasm, we hypothesized that circUBE2K could act as miRNA sponge. RNA interaction prediction tools including miRanda, Circular RNA Interactome and CircBank were used to predict targets of circUBE2K (Fig. 3A). Two miRNAs (miR-516b-5p and miR-1231) were predicted by three different prediction software programs (Fig. 3B). Next, interaction between circUBE2K and miR-516b-5p was confirmed by RNA pulldown assay followed by qRT-PCR (Fig. 3C). By using qRT-PCR, we found a negative correlation between expression level of circUBE2K and miR-516b-5p in BC tissues (Fig. 3D). Co-localization of circUBE2K and miR-516b-5p in cytoplasm was also observed (Fig. 3E). The interaction between circUBE2K and miR-516b-5p was further validated via Ago2 RNA immunoprecipitation (RIP). Higher circUBE2K and miR-516b-5p levels were observed in anti-Ago2 RIP (Fig. 3F), which indicates circUBE2K function as a ceRNA though recruiting Ago2 to interact with miR-516b-5p. To further determine whether miR-516b-5p was a down-stream binding target of circUBE2K, a dual-luciferase reporter assay was performed. Luciferase activity of cells carrying wild type circUBE2K could be lowered by miR-516b-5p mimics. However, luciferase activity was not lowered in cells carrying mutated circUBE2K (Fig. 3G, H). As conclusion, circUBE2K acted as a sponge for miR-516b-5p in BC cells.

### ARHGAP5 was the down-stream target of miR-516b-5p, and it could predict BC prognosis

**Fig. 4:**
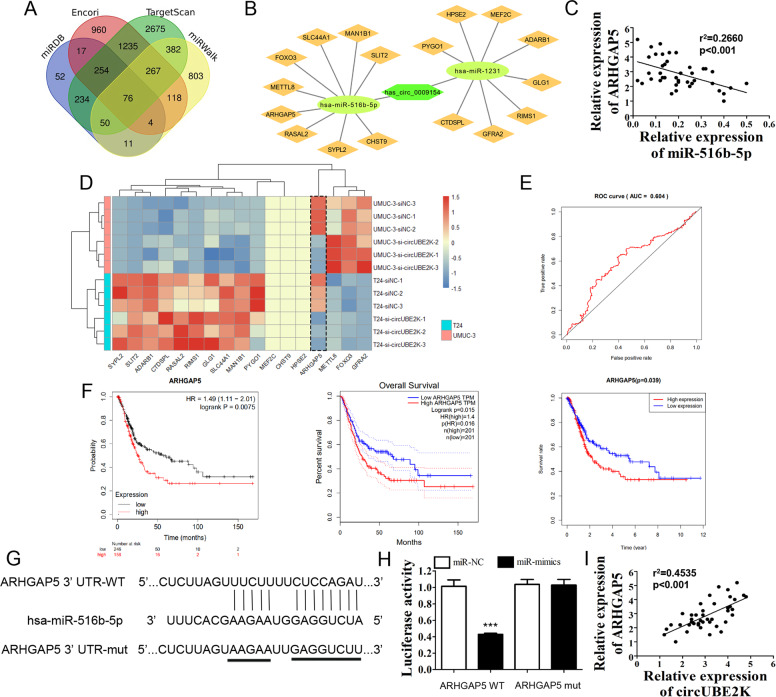
ARHGAP5 was the down-stream target of miR-516b-5p, and it could predict BC prognosis. **A** Venn diagram showed potential down-stream targets of miR-516b-5p predicted by miRDB, Encori, TargetScan and miRWalk. **B** Diagram of ceRNA network showed circUBE2K/miR-516b-5p/ARHGAP5 axis. **C** Scatter plot presented negative correlation between expression level of miR-516b-5p and ARHGAP5 in BC tissues (*n* = 42, *r*^2^ = 0.2660, *P* < 0.001). **D** Potential down-stream gene expression levels were carried out by qRT-PCR. Heatmap illustrated 17 candidate gene expression level in UM-UC-3 and T24 transfected with siNC and si-circUBE2K (Z-Score scaled). **E** Time-dependent receiver operating characteristic (ROC) curve showed efficacy of ARHGAP5 expression level predicting BC 3-year OS. The area under curve (AUC) was 0.604. **F** Kaplan–Meier plots for BC patients’ OS, based on ARHGAP5 expression level (Left: Kaplan-Meier Plotter; Middle: GEPIA; Right: TCGA database). **G** Binding site of ARHGAP5 3′-UTR and miR-516b-5p was predicted by Interactome. The third row showed mutated ARHGAP5 3′-UTR sequence for further dual-luciferase reporter assay. **H** Luciferase activity were measured 3 days after co-transfection with ARHGAP5 (ARHGAP5 WT)/mutated ARHGAP5 (ARHGAP5 MUT) and miRNA mimics negative control (miR-NC)/hsa-miR-516b-5p mimics (miR-mimics) in HEK-293T cells. Error bars indicate SD (^***^P < 0.001 vs. vehicle). **I** Scatter plot presented positive correlation between expression level of circUBE2K and ARHGAP5 in BC tissues (*n* = 42, *r*^2^ = 0.4535, *P* < 0.001).

Four bioinformatics tools were used to predict mRNA 3′-untranslated region (3′-UTR) interaction with miR-516b-5p. Venn diagram showed potential down-stream targets of miR-516b-5p (Fig. 4A). In order to further determined potential genes which exert potent biological effect in bladder cancer particularly, we screened the 76 genes in GEPIA to further select 17 genes which were differential expressed significantly in TCGA bladder cancer cohort to construct the circRNA-miRNA-mRNA network, while miR-1231 regulated genes were seen as negative control (Fig. 4B). qRT-PCR of 42 paired BC tissues showed that relative ARHGAP5 expression was negatively correlated with miR-516b-5p level (Fig. 4C), while following profiling of 17 predicted miRNA regulated genes in T24/UM-UC-3 versus circUBE2K knockdown T24/UM-UC-3 verified si-circUBE2K in BC cells uniquely reduced ARHGAP5 expression (Fig. 4D). Henceforth, we gained interest in the role of ARHGAP5 in BC. Survival data and gene expression data were then exacted from the cancer genome atlas (TCGA). ROC curve showed a well efficacy of ARHGAP5 expression level predicting BC 3-year OS (Fig. 4E). Three survival analysis carried out by online tools including Kaplan-Meier Plotter, GEPIA and TCGA database, consistently showed that patients with higher ARHGAP5 expression level had worse prognosis. Dual-luciferase reporter assay was conducted to verify whether miR-516b-5p could interact with ARHGAP5 mRNA by binding its 3′-UTR. Luciferase activity of cells carrying wild type ARHGAP5 could be lowered by miR-516b-5p mimics. However, luciferase activity was not lowered in cells carrying mutated ARHGAP5 (Fig. 4G, H). Finally, ARHGAP5 expression is positively correlated with circUBE2K level (Fig. 4I). Our results indicate that ARHGAP5, a prognosis indicator of BC, serves as miR-516b-5p’s target, and may act as the effector protein of circUBE2K through a circUBE2K-miR-516-5p-ARHGAP5 regulation mechanism.

### Silencing miR-516b-5p or ARHGAP5 overexpression recovered inhibitory of invasion and proliferation in BC cell as well as RhoA activity causing by circUBE2K knocking down

**Fig. 5:**
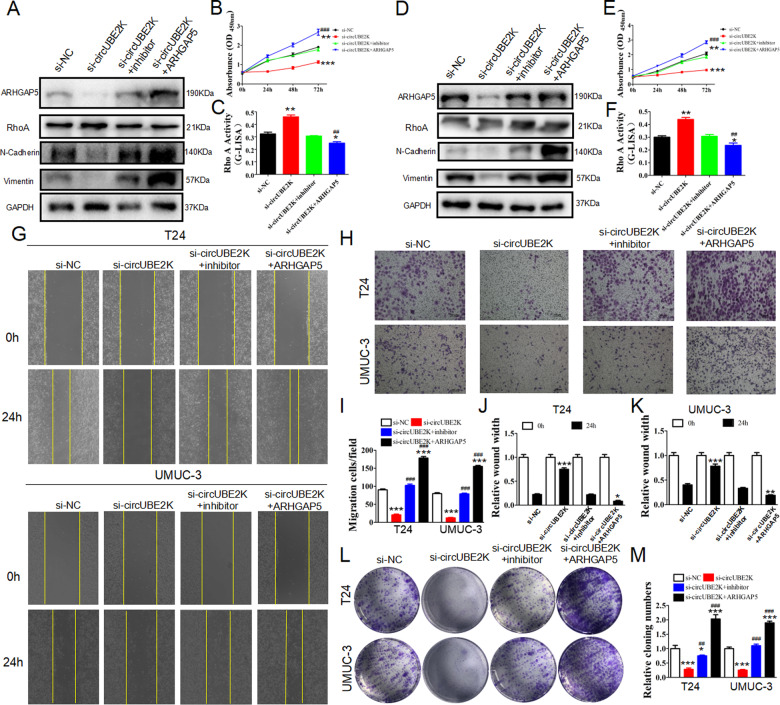
Downregulation miR-516b-5p or ARHGAP5 overexpression restored the proliferation and invasion ability of BC cells after circUBE2K was silenced. T24 and UM-UC-3 cells were transfected with scramble siRNA (si-NC), circUBE2K knockdown siRNA (si-circUBE2K), circUBE2K siRNA together with miRNA inhibitor (si-circUBE2K+inhibitor), circUBE2K siRNA together with ARHGAP5 overexpression vector (si-circUBE2K + ARHGAP5). **A**, **D** Cells were then subjected to western blot analysis anti-ARHGAP5, anti-N-Cadherin, anti-Vimentin and anti-GAPDH. **B**, **E** CCK-8 assays of transfected T24 and UM-UC-3 cells at 0 h, 24 h, 48 h, and 72 h after transfection were performed (^***^P < 0.001, ^**^P < 0.01 versus si-NC, ^###^*P* < 0.001 versus si-circUBE2K). **C**, **F** RhoA G-LISA activation assays were used to evaluate RhoA activity in transfected T24 and UM-UC-3(^**^P < 0.01, ^*^P < 0.05 versus si-NC, ^##^*P* < 0.01 versus si-circUBE2K). **G**, **J**, **K** Wound healing assays at 0 h and 24 h were done to assess cell migration ability (Upper panel: T24-si-NC, T24-si-circUBE2K, T24-si-circUBE2K+inhibitor, T24-si-circUBE2K + ARHGAP5; Lower panel: UM-UC-3-si-NC, UM-UC-3-si-circUBE2K, UM-UC-3-si-circUBE2K+inhibitor, UM-UC-3-si-circUBE2K + ARHGAP5) (^***^P < 0.001, ^**^P < 0.01, ^*^P < 0.05 versus si-NC). **H**, **I** Transwell cell migration assays of transfected T24 and UM-UC-3 were carried out (^***^P < 0.001 versus si-NC, ^###^*P* < 0.001 versus si-circUBE2K). **L**, **M** Colony formation assays of transfected T24 and UM-UC-3 were carried out (^***^P < 0.001, ^*^P < 0.05 versus si-NC, ^###^*P* < 0.001, ^##^*P* < 0.01 versus si-circUBE2K).

To further investigate the biological relationship between circUBE2K, miR-516b-5p, and ARHGAP5, we tested whether silencing miR-516b-5p or ARHGAP5 overexpression could rescue the biological consequence after the circUBE2K knockdown. While ARHGAP5 serves as a RhoA inhibitor and processing cellar function through promoting EMT, western blot illustrated that ARHGAP5 expression and EMT process were inhibited after adding si-circUBE2K. The suppression recovered when miR-516b-5p knockdown restored or ARHGAP5 overexpression in T24 and UM-UC-3 (Fig. 5A, D). CCK-8 assays suggested co-transfected si-circUBE2K and miR-516b-5p inhibitor could partly attenuated the suppressing effect caused by si-circUBE2K, and even restored after ARHGAP5 overexpression (Fig. 5B, E). Moreover, G-LISA assays also confirmed that RhoA activity was up-regulated after circUBE2K suppressing but inhibited after miR-516b-5p inhibition or ARHGAP5 overexpression (Fig. 5C, F). Similar results were observed on BC cell migration (Fig. 5G, J, K) and cell invasion (Fig. 5H, I) in T24 and UM-UC-3, as well as the result of colony formation (Fig. 5L, M). Thus, our data classified that si-circUBE2K-related BC cell inhibitory could be reversed via silencing miR-516b-5p or over-expressing of ARHGAP5 through a RhoA dependent mechanism.

### Overexpressing ARHGAP5 restored proliferation and migration of BC cell and RhoA activity after ectopic expression of miR-516b-5p

**Fig. 6:**
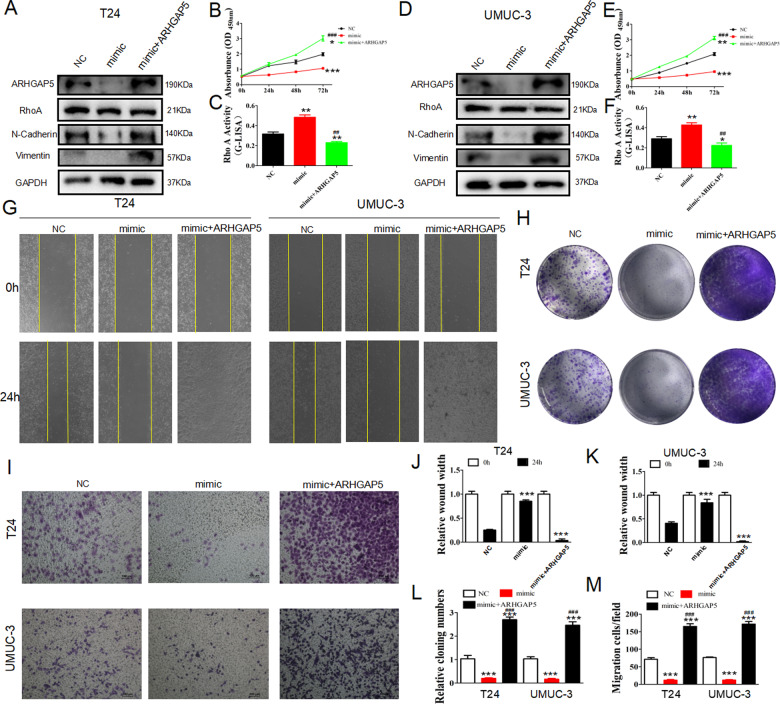
Upregulation of ARHGAP5 rescued the ability of proliferation and invasiveness of BC cells after miR-516b-5p overexpression. T24 and UM-UC-3 cells were transfected with empty vector (NC), hsa-miR-516b-5p mimics (mimic), hsa-miR-516b-5p mimics together with ARHGAP5 overexpression vector (mimic+ARHGAP5). (**A**, **D**) Cells were then subjected to western blot analysis with anti-ARHGAP5, anti-N-Cadherin, anti-Vimentin and anti-GAPDH. GAPDH was used as loading control. (**B**, **E**) CCK-8 assays of transfected T24 and UM-UC-3 cells at 0 h, 24 h, 48 h and 72 h after transfection were performed (^***^P < 0.001,^**^P < 0.01,^*^P < 0.05 versus NC, ^###^*P* < 0.001 versus si-circUBE2K) (C and F) RhoA G-LISA activation assays were used to evaluate RhoA activity in transfected T24 and UM-UC-3 (^**^P < 0.01,^*^P < 0.05 versus NC, ^##^*P* < 0.01 versus si-circUBE2K). (**G**, **J**, **K**) Wound healing assays at 0 h and 24 h were done to assess cell migration ability (Left panel: T24-NC, T24-mimic, T24-mimic+ARHGAP5; Right panel: UM-UC-3-NC, UM-UC-3-mimic, UM-UC-3-mimic+ARHGAP5) (^***^P < 0.001 versus NC). (**H**, **L**) Colony formation assays of transfected T24 and UM-UC-3 were carried out to test cell proliferation potential.(^***^P < 0.001 versus NC, ^###^*P* < 0.001 versus si-circUBE2K) (**I**, **M**) Transwell cell migration assays of transfected T24 and UM-UC-3 were carried out to evaluate cell invasion ability(^***^P < 0.001 versus NC, ^###^*P* < 0.001 versus si-circUBE2K).

To further investigate how miR516b-5p and ARHGAP5 promote BC proliferation and migration, we constructed miRNA mimics to over-express miR-516b-5p. The results indicated that in T24 and UM-UC-3, EMT process would be suppressed by miR-516b-5p but restored by ARHGAP5 (Fig. 6A, D). The proliferation of T24 could also be suppressed by miR-516b-5p, however, reversed under ARHGAP5 overexpression (Fig. 6B). G-Lisa assays indicated that RhoA was activated by miR-516b-5p and inhibited by ARHGAP5(Fig. 6C), similar in UM-UC-3(Fig. 6E, F). According to wound healing (Fig. 6G, J, K) and Transwell assays (Fig. 6I, M), ARHGAP5 could restore the suppression of T24 and UM-UC-3 cells’ migration and invasion treated by miR-516b-5p mimics. Colony formation assays demonstrated that in T24 and UM-UC-3, miR-516b-5p could suppress proliferation while ARHGAP5 could promote it (Fig. 6H, L). Taking together, miR-516b-5p suppressed the progression of BC by inhibiting ARHGAP5. Our results demonstrated that silencing circUBE2K suppressed BC progression via circUBE2K/miR-516b-5p/ARHGAP5 axis, and ARHGAP5 could also suppress RhoA activity to play a vital role in BC progression.

### Silencing circUBE2K inhibited EMT through increasing Rho A activity independent of TGF-β induced P-AKT regulation in bladder cancer cell

**Fig. 7:**
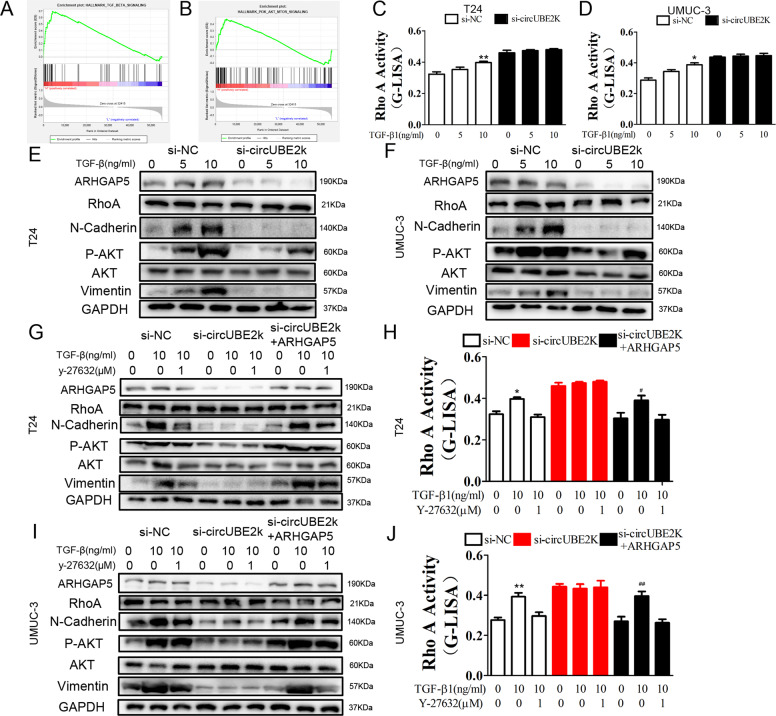
Silencing circUBE2K increased TGF-β stimulated Rho A activity mediated EMT in bladder cancer cell. TGFβ signaling (**A**) and PI3K AKT MTOR signaling (**B**) were significantly enriched in ARHGAP5 highly expressed bladder cancer samples in TCGA cohort through GSEA. RhoA G-LISA activation assays were used to measure RhoA activity in T24 (**C**) and UM-UC-3 (**D**) treated with different concentrations of TGF-β1 (^*^P < 0.05 versus si-NC). T24 (**E**) and UM-UC-3 (**F**) cells transfected with si-NC and si-circUBE2K were treated with different concentrations of TGF-β1, of which cell lysates were then subjected to western blot analysis with anti-ARHGAP5, anti-p-AKT, anti-N-Cadherin, anti-Vimentin and anti-GAPDH. T24 (**G**) and UM-UC-3 (**I**) cells were transfected with si-NC, si-circUBE2K, si-circUBE2K + ARHGAP5 and then treated with or without 10 ng/ml TGF-β1 and 1 µM Y-27632. Cell lysates were then subjected to western blot analysis. Meanwhile, RhoA activity was measured by G-LISA activation assays across T24 (**H**) and UM-UC-3 (**J**) (^**^P < 0.01, ^*^P < 0.05 versus si-NC, ^##^*P* < 0.01, ^#^*P* < 0.05 versus si-circUBE2K + ARHGAP5).

To explore the mechanism involved in circUBE2K regulating EMT, GSEA was applied in TCGA-BLCA. TGF-β signaling (Fig. 7A) and PI3K/AKT/MTOR signaling (Fig. 7B) were significantly enriched in ARHGAP5 highly expressed samples, which indicated circUBE2K/ARHGAP5 regulated BC progression may involve regulated via TGF-β and PI3K/AKT/MTOR related metabolism pathway. RhoA G-LISA activation assays showed that TGF-β1 could increase RhoA activity in a concentration-dependent manner, which could be repressed in condition of circUBE2K knockdown while si-circUBE2K significantly saturated RhoA activity in both T24 cells (Fig. 7C) and UM-UC-3 cells (Fig. 7D), indicating that RhoA and circUBE2K may participate in TGF-β1 induced downstream signaling. To further focus on TGF-β1 induced biological effect and the relationship with RhoA, we examined EMT marker and P-AKT expression level. TGF-β1 induced concentration-dependent EMT marker elevation, together with P-AKT level. RhoA regulated EMT process without affecting P-AKT level under TGF-β, while TGF-β could activate P-AKT in regulating EMT process. Interestingly, circUBE2K knockdown diminished effect of TGF-β1 on EMT without affecting AKT phosphorylation (Fig. 7E, F), which indicates RhoA regulates si-circUBE2K induced EMT inhibitory. We also found that Y-27632(ROCK inhibitor) treatment suppressed TGF-β1 mediated EMT. Knockdown of circUBE2K inhibited both TGF-β1 and Y-27632 effect on EMT, which could be recovered by down-regulating RhoA activity through overexpressing ARHGAP5. Notably, neither circUBE2K knockdown nor ARHGAP5 overexpression significantly affected AKT phosphorylation, indicating that circUBE2K regulates EMT through a non-metabolic way (Fig. 7G, I). Furthermore, we carried out G-LISA RhoA activity assay. As shown in Fig. 7H, J, Y-27632 inhibited TGF-β1 induced elevation of RhoA activity. Y-27632 shows an antagonize effect on TGF-β1 induced RhoA activity, while circUBE2K knockdown or overexpressing ARHGAP5. Our results, together with previous results indicated circUBE2K orchestrate BC progression via circUBE2K/miR-516b-5p/ARHGAP5/ RhoA mediated EMT process.

### Silencing circUBE2K suppressed BC growth and metastasis in vivo which could rescued via miR-516b-5p inhibition or ARHGAP5 overexpression

**Fig. 8:**
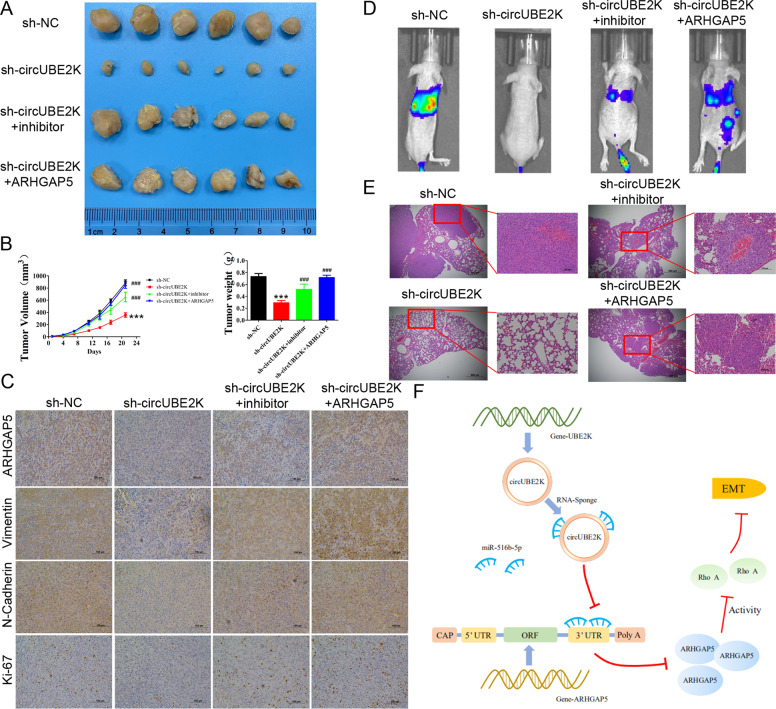
Silencing circUBE2K suppressed BC growth in vivo. **A** Tumor sizes of T24-sh-NC and T24-sh-circUBE2K were shown in the figure. **B** Tumor growth of subcutaneous T24 tumors transfected with sh-NC (closed circles) or sh-circUBE2K (closed square) 21 days after tumor implantation. Average weights of tumors from sh-NC group and sh-circUBE2K group were shown in the histogram. Error bars indicate SD. **C** Expression levels of ARHGAP5, Vimentin, N-Cadherin, and Ki-67 were measured by immunohistochemical staining. Representative views of slides from sh-NC group (Left panel) and sh-circUBE2K (Right panel) were shown. **D** Bioluminescence imaging in mice metastasis model (Left, NC-T24) and circUBE2K knockdown T24 cells (Right, si-circUBE2K-T24). Imaging revealed that silencing circUBE2K reduced metastasis potential of T24 cells. **E** Representative hematoxylin and eosin-stained sections of lung tissues from NC-T24 (Top) and si-circUBE2K-T24 (Bottom) bearing mice. **F** System role of circUBE2K in the oncogenesis scheme of bladder cancer.

To explore whether down-regulated circUBE2K regulates BC growth in vivo, T24 cells transfected with scramble shRNA (sh-NC), shRNA targeting circUBE2K (sh-circUBE2K), shRNA targeting circUBE2K with miR-516b-5p inhibitor (sh-circUBE2K+inhibitor) and shRNA targeting circUBE2K with ARHGAP5 overexpression vector (sh-circUBE2K + ARHGAP5) were injected into nude mice subcutaneously and measured. circUBE2K knockdown significantly reduced the growth rate and weight of BC tumor, which could be rescued via miR-516b-5p inhibition or ARHGAP5 overexpression (Fig. 8A, B). In compliance with our previous findings, IHC analysis revealed that the expression of ARHGAP5, EMT marker (Vimentin and N-Cadherin), cell proliferation marker (Ki-67) was inhibited by silencing circUBE2K and recovered after silencing miR-516b-5p or up-regulation of ARHGAP5 (Fig. 8C). Furthermore, bioluminescence imaging revealed that silencing circUBE2K reduced metastasis potential of T24 cells (Fig. 8D). In line with the micropathological results, hematoxylin and eosin-stained tissues showed that sh-circUBE2K-T24 cells formed up fewer nodules and smaller lesion areas in the lungs, which miR-516b-5p inhibition or ARHGAP5 overexpression could partially recover the metastasis ability of T24 cells (Fig. 8E).

Taken together, the system role of circUBE2K in the oncogenesis scheme of BC showed in Fig. 8F.

## Discussion

In this work, we first confirmed circUBE2K played an important tumor-promoting role in BC and found that high expression of circUBE2K was positively correlated with poor overall survival in BC patients. Local progression and metastasis are the leading causes of the resultant mortality in BC patients [26], which urged us to further investigate the molecular hallmarks of invasiveness and metastasis. Bioluminescence imaging assay of lung metastasis mouse xenograft model revealed that silencing circUBE2K did reduce the tumor metastasis. Moreover, G-LISA and western blot revealed that RhoA activity was negatively regulated by ARHGAP5 and circUBE2K. Nevertheless, we further demonstrated ARHGAP5 could promote BC metastasis and invasion by negatively regulate RhoA activity through non-metabolic EMT pathway.

Circular RNA, a novel type of noncoding RNAs, formed a covalently closed continuous loop and played a crucial role in the process of gene transcription and translation [27]. Several circRNAs with tumor-promoting functions have been wildly characterized, such as circSEPT9 [28], circFOXM1 [29], circMAST1 [30]. Currently, competitive endogenous RNA viewpoint is the most in-depth research and a widely recognized mechanism of action [31]. Our previous studies have identified that circRGNEF work as miRNA sponge and thus promote BC progression via miR-548/KIFC2 axis [12]. Here, we verified an innovative and tumor-promoting circUBE2K by high-throughput sequencing and determined its downstream miR-516b-5p, which acts as a tumor suppressor in esophageal squamous cell carcinoma and colorectal cancer [32, 33]. We first declared its anti-tumor effects in BC and found downstream protein ARHGAP5 by combining bioinformatic and RNA-RNA pulldown analysis.

ARHGAP5, member of the Rho family of small GTPases, which may mediate cytoskeleton changes by stimulating the hydrolysis of bound GTP [34]. As previous described, ARHGAP5 could work as an oncogene to promote tumor proliferation and migration in hepatocellular carcinoma [35] and gastric cancer [36]. Following previous work, ARHGAP5 could regulate RhoA activity and EMT process [37]. Ras homolog gene family member A (RhoA) is a small GTPase protein, which can regulate cell morphology, motility, and cell adhesion [38]. While several mRNA regulated via circRNA has been identified, there is still no Rho GTPase regulated through ceRNA regulation of circRNA. Therefore, we assumed that RhoA, indirectly regulated by circUBE2K, played a crucial role in tumor migration and metastasis. Indeed, G-LISA assays showed that activity of RhoA was improved under circUBE2K silent but reversed after adding ARHGAP5. Thus, we can summarize that RhoA activity up-regulated could suppress tumor effectively which is largely consistent with prior studies [39]. According to the GSEA analysis, we predicted that TGF-β-signaling and PI3K-AKT-MTOR-signaling may act as relative pathway in this process. Thus, we detected hallmarks of EMT and metabolism after RhoA activator TGF-β, RhoA/ROCK inhibitor Y-27632 separately added. Based on the results, we observed that TGF-β could promote N-Cadherin, Vimentin, and P-AKT expression. However, N-Cadherin and Vimentin expression were suppressed while P-AKT was unregulated by RhoA inhibitor Y-27632. What’s more, in the condition of circUBE2K silent, RhoA activity was upregulated under TGF-β added but reversed by Y-27632. Taken together, we can summarize that BC migration and metastasis are relevant to the circUBE2K-regulated RhoA, and mediate non-metabolic EMT pathway.

In conclusion, our findings report that circUBE2K expression is significantly overexpressed in BC tissues and that high levels of circUBE2K expression correlate with a poor overall survival for BC patients. As a sponge of miR-516b-5p, circUBE2K could up-regulate expression of target gene ARHGAP5, and thus promote EMT and exert tumor stimulating effect on proliferation and metastasis. Collectively, the circUBE2K/miR-516b-5p/ARHGAP5 network may be a potential target for BC treatment and deserves further research.

## Supplementary information

supplementary table 1

supplementary Figure 1 and 2

Supplementary material
