# Correction to: High-throughput sequencing identified circular RNA circUBE2K mediating RhoA associated bladder cancer phenotype via regulation of miR-516b-5p/ARHGAP5 axis

**DOI:** 10.1038/s41419-022-05509-x

**Published:** 2022-12-22

**Authors:** Chen Yang, Zezhong Mou, Siqi Wu, Yuxi Ou, Zheyu Zhang, Xinan Chen, Xiyu Dai, Chenyang Xu, Shanhua Mao, Haowen Jiang

**Affiliations:** 1 Department of Urology, Huashan Hospital, Fudan University, Shanghai, China; 2 Fudan Institute of Urology, Huashan Hospital, Fudan University, Shanghai, China; 3 National Clinical Research Center for Aging and Medicine, Fudan University, Shanghai, China

**Keywords:** Non-coding RNAs, Bladder cancer

Correction to: *Cell Death and Disease* (2021) **12**:719 10.1038/s41419-021-03977-1, published online 20 July 2021

The original version of this article contained a mistake. In figure 2E, the “UMUC-3 NC” group of cell cycle was wrong. In figure 2F, the “NC” group should be corrected as “si-NC” in a more appropriate description. In figure 5G “UMUC-3 si-NC 0h” and figure 6G “UMUC-3 NC 0h” was placed with wrong figures. The authors corrected all mistakes with the corrected figs. 2, 5 and 6 in this correction. The original article has been corrected.

**Figure 1:**
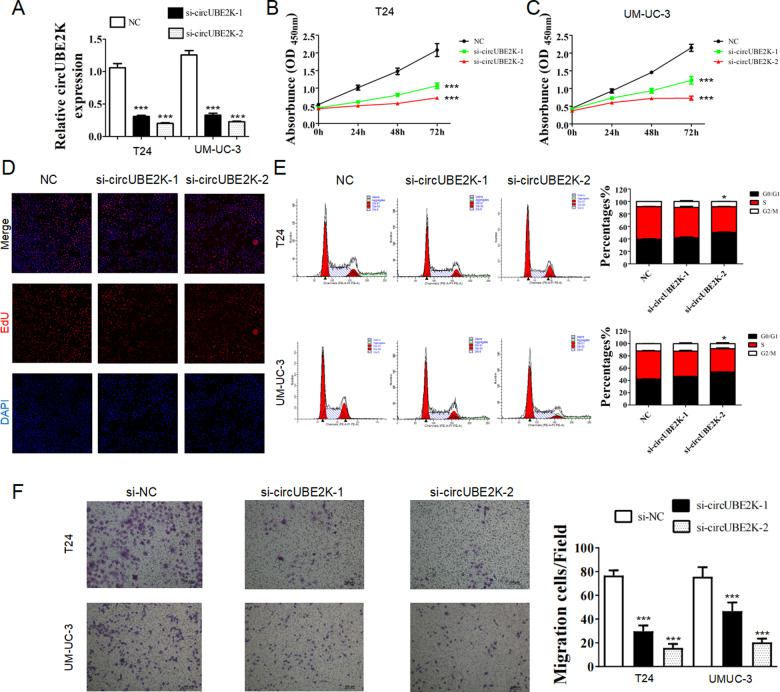
Fig. 2

**Figure 2:**
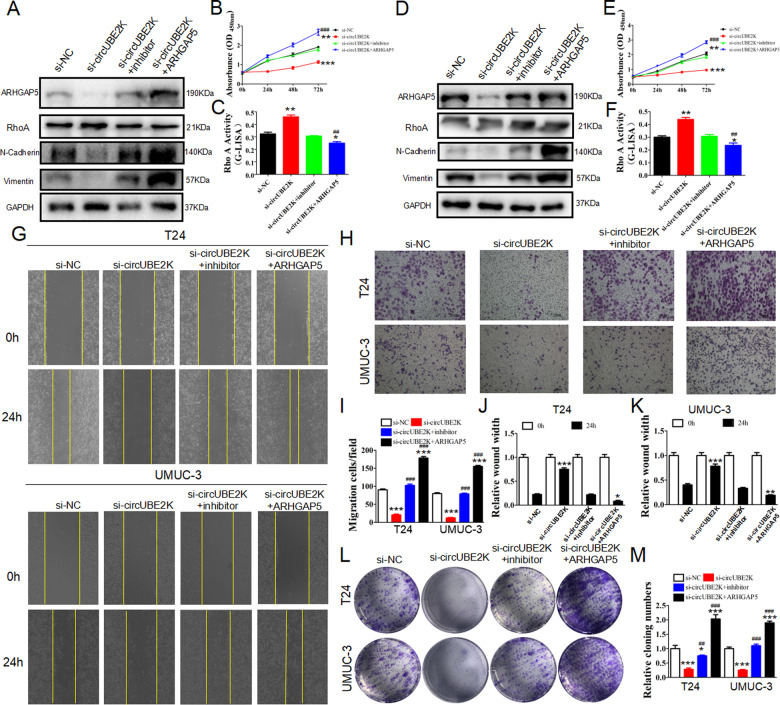
Fig. 5

**Figure 3:**
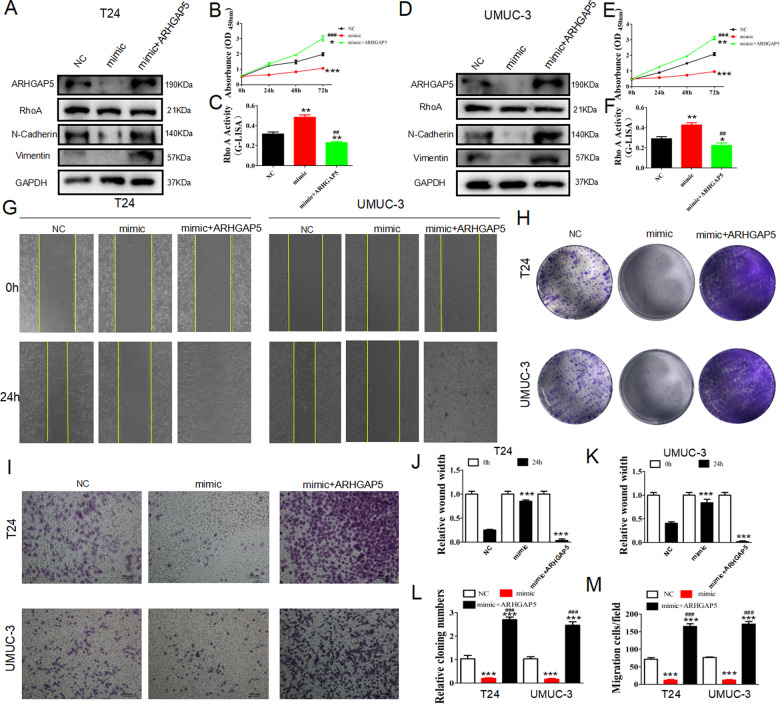
Fig. 6

