# Supplementary material for: High-throughput sequencing identified circular RNA circUBE2K mediating RhoA associated bladder cancer phenotype via regulation of miR-516b-5p/ARHGAP5 axis
Source: Cell Death Dis. 2021 Jul 20;12(8):719. doi: 10.1038/s41419-021-03977-1 (PMC8292476; doi:10.1038/s41419-021-03977-1)
Supplement: Supplementary file 1 — supplementary table 1 [file 41419_2021_3977_MOESM1_ESM.doc]

**Table 1** Relationship between the expression levels of circUBE2K and clinicopathological features in bladder cancer

| Characteristics | No. (%) | circUBE2K expression | | |
| --- | --- | --- | --- | --- |
|  |  | Low (%) | High (%) | *P*-value |
| **Gender** | | | | |
| Male | 81 (90.0) | 25 (30.9) | 56 (69.1) | 1.000 |
| Female | 9 (10.0) | 3 (33.3) | 6 (66.7) |  |
| **Age** | | | | |
| <65 | 56 (62.2) | 15(26.8) | 41(73.2) | 0.348 |
| ≥65 | 34 (37.8) | 13(38.2) | 21(70.6) |  |
| **Tumour size** | | | | |
| <3 cm | 38 (42.2) | 18 (47.4) | 20 (52.6) | 0.006 |
| ≥3 cm | 52 (57.8) | 10 (19.2) | 42 (80.8) |  |
| **Clinical stage** | | | | |
| Ta-T1 | 44 (48.9) | 19 (43.2) | 25 (56.8) | 0.022 |
| T2-T4 | 46 (51.1) | 9 (19.6) | 37 (80.4) |  |
| **Grade** | | | | |
| Low | 51 (56.7) | 22 (43.1) | 29 (56.9) | 0.006 |
| High | 39 (43.3) | 6 (15.4) | 33 (84.6) |  |
| **Lymphatic metastasis** | | | | |
| Yes | 22 (24.4) | 1 (4.5) | 21 (95.5) | 0.001 |
| No | 68 (75.6) | 27 (39.7) | 41 (60.3) |  |
| **Muscle invasion** | | | | |
| NMIBC | 61 (67.8) | 26 (42.6) | 35 (57.4) | 0.001 |
| MIBC | 29 (32.2) | 2 (6.9) | 27 (93.1) |  |
| Total | 90 | 28 | 62 |  |
