# Supplementary material for: High-throughput sequencing identified circular RNA circUBE2K mediating RhoA associated bladder cancer phenotype via regulation of miR-516b-5p/ARHGAP5 axis
Source: Cell Death Dis. 2021 Jul 20;12(8):719. doi: 10.1038/s41419-021-03977-1 (PMC8292476; doi:10.1038/s41419-021-03977-1)
Supplement: Supplementary file 2 — supplementary Figure 1 and 2 [file 41419_2021_3977_MOESM2_ESM.docx]

**
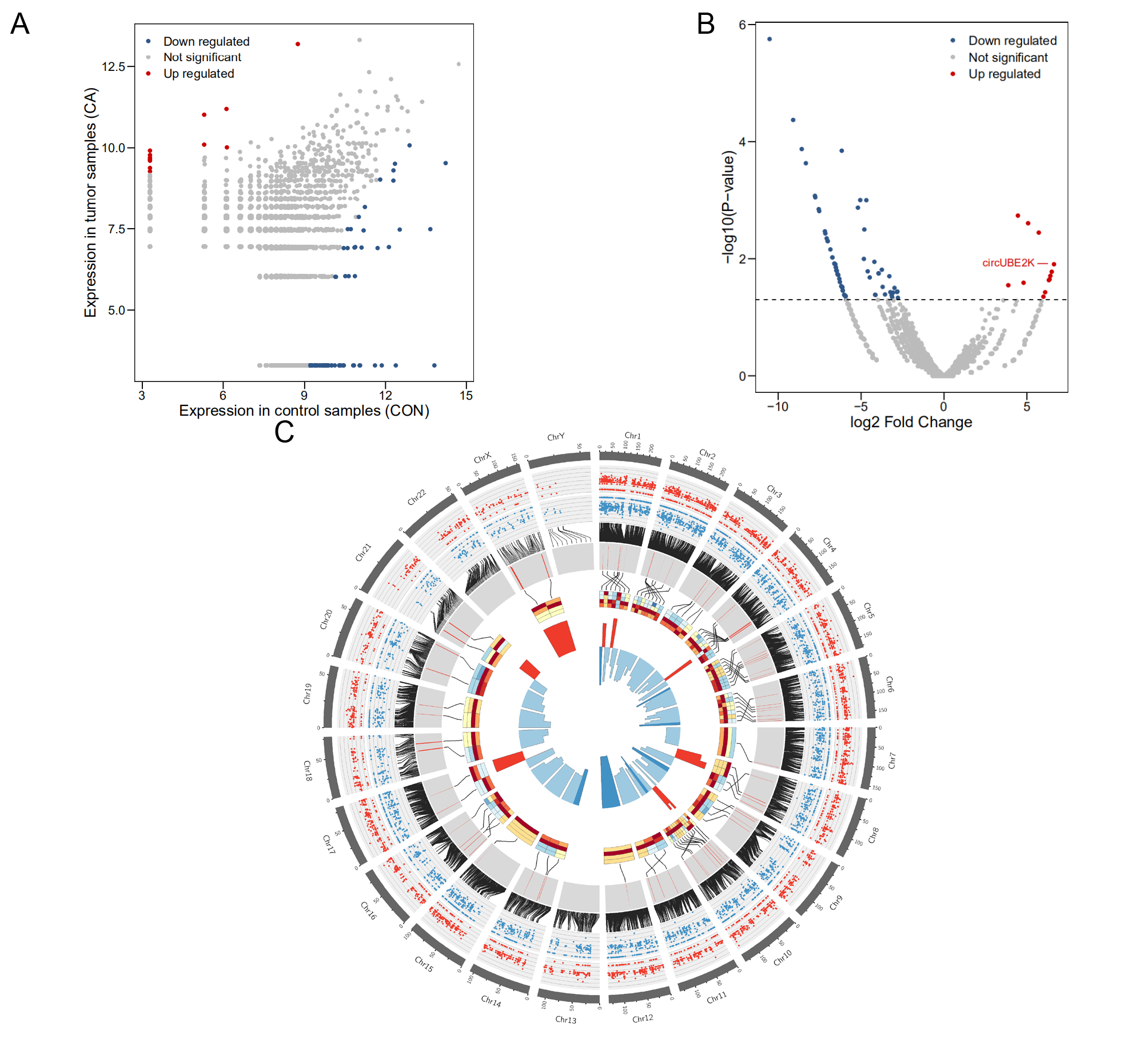
**

**Supplementary Figure 1 Identification of has-circ-0009154 (circUBE2K) as a novel circular RNA in bladder cancer through high-throughput sequencing.**

(A) Total differential expression of circRNAs in two pairs of bladder cancer. (B) Volcano plots illustrate 12 upregulated circRNAs and 78 downregulated circRNAs in bladder cancer among significantly differential expressed circRNAs relative to normal tissues. (C) Genomic location and differential expression pattern of high-throughput sequencing of two pairs of bladder cancer through Circos (http://circos.ca)


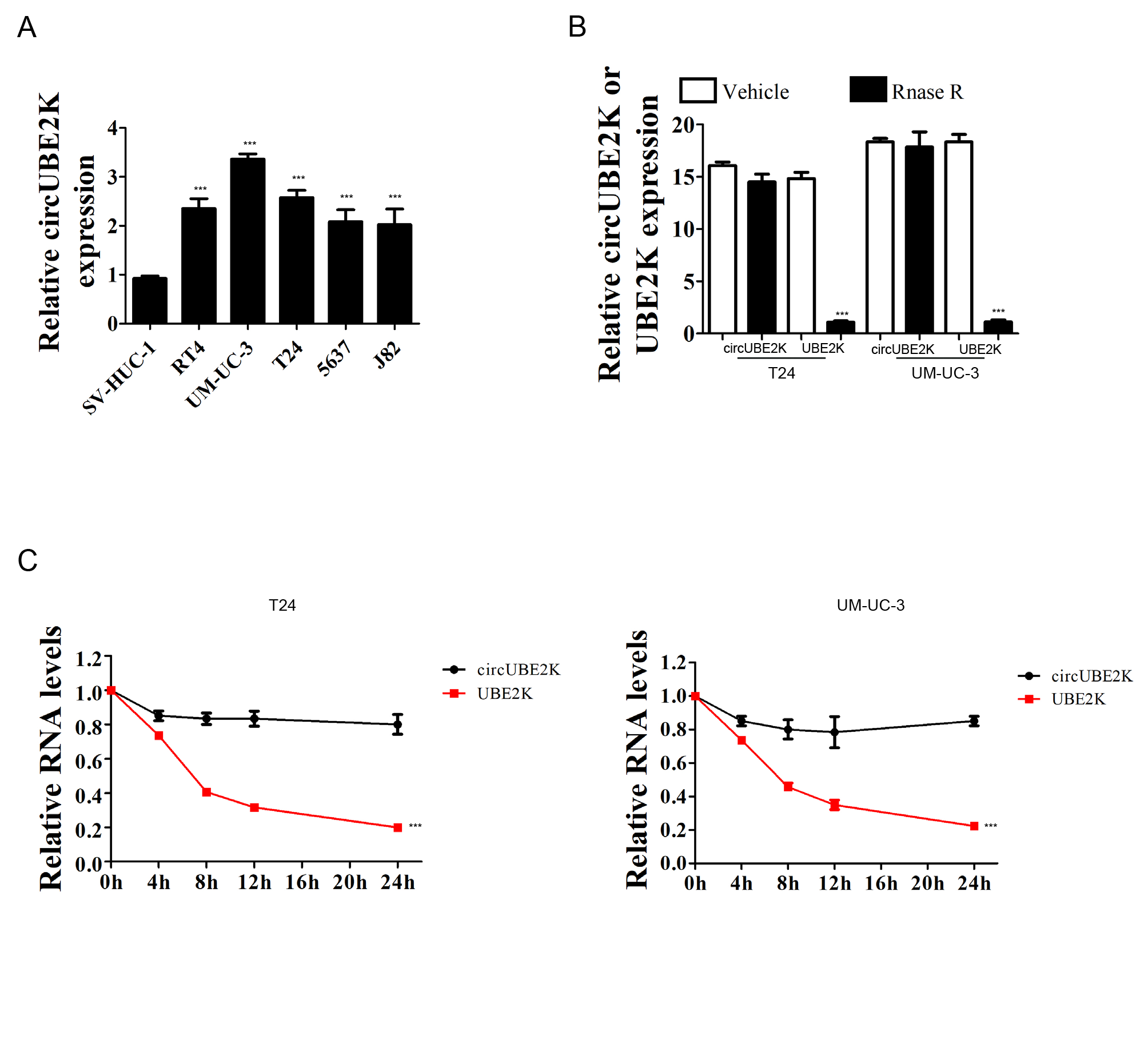


**Supplementary Figure 2** **Expression of circUBE2K was upregulated and stable in BC cell lines.**

(A) qRT-PCR showed circUBE2K was overexpressed in five bladder transitional cell carcinoma cell lines (RT4, UM-UC-3, T24, 5637, J82) compared to immortalized uroepithelium cell line (SV-HUC-1). Error bars indicate SD (^***^P <0.001, ^**^P<0.01, ^*^P<0.05 versus SV-HUC-1). (B) Total RNA from T24 and UM-UC-3 cells was treated with or without RNase R. Relative expression of circUBE2K and UBE2K mRNA was measured by qRT-PCR. Error bars indicate SD (^***^P<0.001 versus vehicle cell). (C) Relative RNA abundance of circUBE2K and UBE2K treated with 2ug/ml Actinomycin D at indicated time points with 3 independent replication samples in T24 and UMUC-3. (D) Fluorescent in situ hybridization (FISH) for circUBE2K (Green) and DAPI (Blue) in 90 pairs of bladder cancer tissue microarray. circUBE2K is up regulated in BC tissue (Right panel) comparing to paired normal tissues (Left panel). Also, circUBE2K locates in BC cell cytoplasm. Representative area was selected.
