# Supplementary material for: High-throughput sequencing identified circular RNA circUBE2K mediating RhoA associated bladder cancer phenotype via regulation of miR-516b-5p/ARHGAP5 axis
Source: Cell Death Dis. 2021 Jul 20;12(8):719. doi: 10.1038/s41419-021-03977-1 (PMC8292476; doi:10.1038/s41419-021-03977-1)
Supplement: Supplementary file 3 — Supplementary material [file 41419_2021_3977_MOESM3_ESM.docx]

**Supplementary methods and materials**

The sequence of primer targeting GAPDH were:

Forward, 5’--3’; AATGGACAACTGGTCGTGGAC

Reverse, 5’--3’; CCCTCCAGGGGATCTGTTTG

The sequence of primer targeting UBE2K were:

Forward, 5’--3’; GTTCCGTCACAGGGGCTATTT

Reverse, 5’--3’; AATACCGTGCGGAGAGTCATT

The sequence of primer targeting back-splicing region of circUBE2K were:

Forward, 5’--3’; AGGTCCTGCTATTTCTCC

Reverse, 5’--3’; AAGGTCCGGTTTATCACT

The sequence of primer targeting hsa-miR-516b-5p were:

Forward, 5’--3’; ACACTCCAGCTGGGATCTGGAGGTAAGAAG

The sequence of primer targeting hsa-miR-1231 were:

Forward, 5’--3’; ACACTCCAGCTGGGGTGTCTGGGCGGAC

miRNA reverse primer: TGGTGTCGTGGAGTCG

The sequence of primer targeting ARHGAP5 were:

Forward, 5’--3’; AGGGAAGCTCAACGTAGATGG

Reverse, 5’--3’; ATGATCCACGCATTCATCACAT

The sequence of primer targeting SYPL2 were:

Forward, 5’--3’; CAGTGGCTCTTTGCTATTTTCGC

Reverse, 5’--3’; CTTGGCTTCGTTGTTGCAGC

The sequence of primer targeting SLIT2 were:

Forward, 5’--3’; GCGAAGCTATACAGGCTTGAT

Reverse, 5’--3’; TGCAGTCGAAAAGTCCTAAGTTT

The sequence of primer targeting ADARB1 were:

Forward, 5’--3’; GTGAAGGAAAACCGCAATCTGG

Reverse, 5’--3’; CAGGAGTGTGTACTGCAAACC

The sequence of primer targeting CTDSPL were:

Forward, 5’--3’; TCCCCTGCCTCATACATCTTC

Reverse, 5’--3’; CCGTGTCCGTCATGTCATCG

The sequence of primer targeting RASAL2 were:

Forward, 5’--3’; AGCAGAAAGGTCCCCTCGTAG

Reverse, 5’--3’; AGGGTGAGGTATTTGCAGTGT

The sequence of primer targeting RIMS1 were:

Forward, 5’--3’; TGGAAGTCATTAGAGCACGAAGC

Reverse, 5’--3’; CCCAGACAATCACCTGAAGAACT

The sequence of primer targeting GLG1 were:

Forward, 5’--3’; CCAAGATGACGGCCATCATTT

Reverse, 5’--3’; AGCCGAATACTGCCACATTTC

The sequence of primer targeting SLC44A1 were:

Forward, 5’--3’; AAATGGTTCAGCCCTATGTAGC

Reverse, 5’--3’; TCGCTGGAACTGGTAGTTTGG

The sequence of primer targeting MAN1B1 were:

Forward, 5’--3’; CTCTCGGTTCCTCTCAGTCG

Reverse, 5’--3’; GTGGGTACATGACTACAGTGGT

The sequence of primer targeting PYGO1 were:

Forward, 5’--3’; CAGCTCCCGCTTACAAAGTTT

Reverse, 5’--3’; GGTCCCTGTGTATTTGCCTTG

The sequence of primer targeting MEF2C were:

Forward, 5’--3’; CCAACTTCGAGATGCCAGTCT

Reverse, 5’--3’; GTCGATGTGTTACACCAGGAG

The sequence of primer targeting CHST9 were:

Forward, 5’--3’; ATCCAGGAACATATCACCAACCA

Reverse, 5’--3’; CAGTCTTAGCTCCTTGACGTTTT

The sequence of primer targeting HPSE2 were:

Forward, 5’--3’; ATCGCCCTCCTAGATGGATTC

Reverse, 5’--3’; TCAGAGAGTGTGTCTAACAGGC

The sequence of primer targeting METTL6 were:

Forward, 5’--3’; CAGGCAAGGATTCTCACCTCT

Reverse, 5’--3’; TGGTCCAGTGTCTGTCTTTGA

The sequence of primer targeting FOXO3 were:

Forward, 5’--3’; CGGACAAACGGCTCACTCT

Reverse, 5’--3’; GGACCCGCATGAATCGACTAT

The sequence of primer targeting GFRA2 were:

Forward, 5’--3’; TTGTCGAGCCTCCTACCAGAC

Reverse, 5’--3’; GCCAGCATAAGAGCCCAGA

Sequence of siRNA and miRNA mimics were provided below.

si-NC sense: UUCUCCGAACGUGUCACGUTT

Si-NC antisens: ACGUGACACGUUCGGAGAATT

si-circUBE2K-1 sense: GAAAGAUCAAUGACGAGCATT

si-circUBE2K-1 antisens: UGCUCGUCAUUGAUCUUUCTT

si-circUBE2K-2 sense: AUCAAUGACGAGCAAAAAUTT

si-circUBE2K-2 antisens: AUUUUUGCUCGUCAUUGAUTT

si-circUBE2K-3 sense: AAUGACGAGCAAAAAUCAATT

si-circUBE2K-3 antisens: UUGAUUUUUGCUCGUCAUUTT

miR-NC inhibitor: GGGACAUGGUGGCUUCUUGUCG;

miR-516b-5p inhibitor: AAAGUGCUUCUUACCUCCAGAU;

miR-NC mimics: CGCGAAGAACGGAGGAUGUGGG;

miR-516b-5p mimics: AUCUGG AGGUAAGAAGCACUUU.
